# Stitching gene fragments with a network matching algorithm improves gene assembly for metagenomics

**DOI:** 10.1093/bioinformatics/bts388

**Published:** 2012-09-03

**Authors:** Yu-Wei Wu, Mina Rho, Thomas G. Doak, Yuzhen Ye

**Affiliations:** ^1^School of Informatics and Computing; ^2^Department of Biology, Indiana University, Bloomington, IN 47405, USA

## Abstract

**Motivation:** One of the difficulties in metagenomic assembly is that homologous genes from evolutionarily closely related species may behave like repeats and confuse assemblers. As a result, small contigs, each representing a short gene fragment, instead of complete genes, may be reported by an assembler. This further complicates annotation of metagenomic datasets, as annotation tools (such as gene predictors or similarity search tools) typically perform poorly on configs encoding short gene fragments.

**Results:** We present a novel way of using the de Bruijn graph assembly of metagenomes to improve the assembly of genes. A network matching algorithm is proposed for matching the de Bruijn graph of contigs against reference genes, to derive ‘gene paths’ in the graph (sequences of contigs containing gene fragments) that have the highest similarities to known genes, allowing gene fragments contained in multiple contigs to be connected to form more complete (or intact) genes. Tests on simulated and real datasets show that our approach (called GeneStitch) is able to significantly improve the assembly of genes from metagenomic sequences, by connecting contigs with the guidance of homologous genes—information that is orthogonal to the sequencing reads. We note that the improvement of gene assembly can be observed even when only distantly related genes are available as the reference. We further propose to use ‘gene graphs’ to represent the assembly of reads from homologous genes and discuss potential applications of gene graphs to improving functional annotation for metagenomics.

**Availability:** The tools are available as open source for download at http://omics.informatics.indiana.edu/GeneStitch

**Contact:** yye@indiana.edu

## 1 INTRODUCTION

Metagenomics, also called environmental sequencing, is the study of microbial genomes sampled directly from the environment. We are seeing more metagenomics projects than ever before, due to (i) advances in next-generation sequencing (NGS) technology, such as Roche/454 (Margulies *et al.*, 2005) and Illumina/Solexa (Bentley, 2006); (ii) the fact that only a few species can be cultured and studied using conventional microbiological techniques (Schloss and Handelsman, 2005) and (iii) many studies that have shown the impact of the ‘microbiome’ (i.e. the entire set of genomes in a microbial community) on almost every aspect of life on Earth [e.g. microbes residing in the human body encode far more genes than the genes encoded by the human genome (Gill *et al.*, 2006)]. Metagenomics has been applied to many studies of natural environments (Venter *et al.*, 2004; Tyson *et al.*, 2004) as well as human and animal associated microbiomes (Hess *et al.*, 2011; Peterson *et al.*, 2009; Qin *et al.*, 2010; Turnbaugh *et al.*, 2006), providing an unprecedented opportunity to gain knowledge about the vast majority of uncultured microbial species.

One of the first steps to analyzing metagenomic sequences is to assemble the reads. For example, reads sequenced from the Acid Mine Drainage dataset yielded two near-complete and three partial genomes (Tyson *et al.*, 2004). This, however, is a very simple bacterial community; assembling sequences sampled from most whole microbial communities remains a challenging problem (Pop, 2009). Since most metagenomic sequences are obtained using NGS technology, the traditional ‘overlap-layout-consensus’approach may not be realistic due to the short reads. On the other hand, the de Bruijn graph approach (Compeau *et al.*, 2011), which breaks the reads into *k*-mers and then constructs a de Bruijn graph on these *k*-mers, is difficult because of the mixture of genomic sequences from many species and the higher rate of NGS sequencing errors. As a result, it is difficult to assemble complete genomes from metagenomic sequences, even when the community structure is simple—for example, the Acid Mine Drainage dataset (Tyson *et al.*, 2004) contains merely five species, but yields only two nearly complete genomes.

One of the characteristics of de Bruijn graph-based assemblers is that the resulting graph is usually very tangled, especially when sequencing errors exist. This greatly impedes the formation of long contigs, because the branches cannot be resolved. Moreover, *k*-mers from different regions or even from different species may be connected together, which further complicates the structure of the de Bruijn graph. As a result, many short contigs will be reported, which are often insufficient for downstream analysis, such as *ab initio* gene prediction in these short contigs (Hoff, 2009), or homology searches of the contigs (Wommack *et al.*, 2008). For instance, the MetaHIT consortium only considered contigs of length >500 bp, which represented only 42.7% of the sequencing reads (Qin *et al.*, 2010).

Salzberg *et al.* proposed a gene-boosted assembly approach to improve assembly quality, which used proteins from reference genomes to recruit sequencing reads to fill in the gaps between contigs (2008). Combining this approach with several other strategies, they successfully produced 76 contigs from 8 27 900 33 bp reads obtained from *Pseudomonas aeruginosa* PAb1, with the largest contig being 512 638 bp. They also demonstrated that most of the genes in a newly sequenced bacterial strain can be assembled using the genome of another strain of the same species as the reference, using gene-boosted assembly. This approach, however, was only applied to single genome assembly problems. Metagenome assembly is more difficult, because of the presence of homologous genes from multiple species in the same community that may behave like repeats for assemblers. Hence, the success of the approach relies on the utilization of a closely related genome (e.g. the genome of the same species but a different strain), which may not be available in metagenomics, which aims to study un-cultured microbial species in natural habitats.

Here, we present GeneStitch to infer gene paths (sequences of contigs), each of which represents a gene or a gene fragment, in the tangled de Bruijn graph resulted from *de novo* assembly of metagenomic reads, using a network matching algorithm. Given a reference gene sequence, GeneStitch searches for a path in the de Bruijn graph that is most similar to the given reference gene. Assuming that the gene paths found by GeneStitch consist of reads most likely sampled from a real gene, we can assemble genes in a metagenomic dataset by using known homologous genes as references. When prior knowledge of the species composition and gene contents of the sequenced metagenome is unavailable, we can use as many reference gene sequences as possible (e.g. the entire set of genes from all available microbial genomes) to guide the inference of gene paths.

One challenge of inferring gene paths is the separation of very similar genes in a metagenome. The gene paths inferred from GeneStitch may overlap substantially with each other, because homologous genes will share identical regions. Instead of attempting to separate these individual genes (with the risk of introducing misassemblies), we propose to merge these paths into ‘gene graphs’, each of which is a subgraph of the de Bruijn graph that contains reads from the same gene family (homologous genes). We argue that such gene graphs may be considered as single units for downstream analysis of metagenomes, for example for functional predictions by similarity search.

We test our approach on simulated single-genome and metagenomic datasets, and a mock dataset (Morgan *et al.*, 2010), which consists of real sequencing reads from an artificial community of 10 already-sequenced genomes. The results show that we are able to generate more complete genes by applying GeneStitch, and most of the gene graphs consist only of contigs from homologous genes.

## 2 METHODS

We formulate the inference of gene paths from a de Bruijn graph as a problem of aligning the graph against a set of reference genes, aiming to derive—in the graph—paths of sequence blocks (or contigs) that are most similar to the reference genes; each path represents a gene or a gene fragment that contains shorter gene fragments. Computationally, this problem is equivalent to the ‘network matching problem’ (to find the best alignment between a graph and a sequence, or between two graphs), which has been applied in computational biology; examples are (i) the spliced alignment problem for eukaryotic gene prediction considering all potential exon predictions (Gelfand *et al.*, 1996) and (ii) protein sequence alignments considering all potential secondary structural predictions (Ye *et al.*, 2003). The network matching problem can be solved efficiently by a dynamic programing algorithm that searches for the set of connected blocks with the highest similarity to the reference sequence, without exploring all possible paths through the blocks (which would be exponential in the number of blocks).

### 2.1 Network matching algorithm

**Fig. 1.**
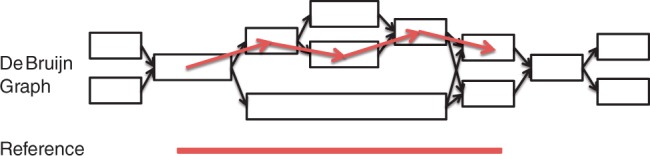
Alignment between a de Bruijn graph and a reference sequence. Blocks in the de Bruijn graph represent nodes, and black arrowheads represent the directed edges that connect nodes with overlapping *k* − 1 mers. Typically, a de Bruijn graph-based assembler will output each of the nodes as a contig. Red arrowheads constitute the optimal path of the nodes that aligns with the reference sequence derived by the network matching algorithm

Consider a set of contigs (*C*_1_,···, *C_n_*) and a de Bruijn graph *G*,^1^ in which each node represents a contig, and a directed edge is connected between two nodes if these two contigs share *k* − 1 nucleotides (*k* is a pre-defined number, e.g. *k* = 30). Our goal is to find the optimal local alignment between the contigs (sequence blocks) and a reference sequence *T* = *t*_1_ ···*t_m_*, as illustrated in Figure 1.

The network matching problem can be solved using a dynamic programming algorithm in polynomial time. Let *S*(*i*,*j*,*k*) be the optimal alignment score between all possible paths ending at position *i* of contig *k* in the input de Bruijn graph and the prefix of the input reference sequence ending at position *j* (i.e., *t*_1_*t*_2_ ···*t_j_*). For each contig *C_k_*, we denote its first letter as *first*(*k*) and its last letter as *last*(*k*). A path in the de Bruijn graph can start from any contig and will contain at least one contig, but must strictly follow the de Bruijn graph structure, where two contigs *C_l_* and *C_k_* can be connected only if a directed edge goes from *C_l_* to *C_k_* (denoted by *C_l_ → C_k_*). Let *E*(*k*)= {*l* : *C_l_ → C_k_* } be the set of contigs that are connected to contig *k*. Our network matching algorithm first computes a dynamic programming matrix to record the optimal alignment scores for 1 ≤ *i* ≤ last(*k*), 1≤ *j*≤ *m*, and 1≤ *k* ≤ *n* (*n* is the total number of contigs). *S*(*i*,*j*,*k*) can be computed recursively as
(1)
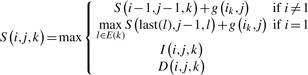

where *i* = 1 indicates it is the first nucleotide in contig *k*, and *g*(*i_k_*,*j*) is the scoring function for matching the nucleotide at position *i* in contig *k* and the nucleotide at position *j* of the input reference sequence: *g*(*i_k_*, *j*)= Δ match if the two nucleotides are the same; otherwise *g*(*i_k_*, *j*)= Δ mismatch (Δ match and Δ mismatch are two preset parameters). *I*(*i*,*j*,*k*) and *D*(*i*,*j*,*k*) are the optimal alignment scores between the paths of the de Bruijn graph (ending at position *i* in contig *k*) and the prefix of the input reference sequence (ending at position *j*), ending with insertion and deletion in the alignment, respectively. The recursive definitions of *I*(*i*,*j*,*k*) and *D*(*i*,*j*,*k*) are as follows:
(2)
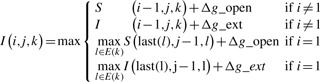

(3)
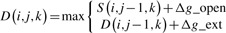

where Δ*g*_open and Δ*g*_ext are penalties for opening and extending gaps, respectively (affine gap penalty is used in our network matching algorithm).

For initialization, *S*(*i*,0,*k*), *S*(0,*j*,*k*), *I*(*i*,0,*k*), *I*(0,*j*,*k*), *D*(*i*,0,*k*) and *D*(0,*j*,*k*) are all set to 0 for all *i*, *j* and *k*.

Once we are done filling in the matrix, we use a traceback procedure to find the best local alignment between the de Bruijn graph and the reference sequence. We first find the maximum score in the dynamic programming matrix and then trace back from that corresponding cell until we reach a score of 0 to find the path of the contigs (which we call a gene path) that leads to the best alignment. We also retrieve the gene sequence by concatenating the nucleotide sequences of the contigs in the path. Since two nodes connected by an edge in a de Bruijn graph overlap in *k* − 1 nucleotides, we need to exclude one redundant copy of the *k* − 1 nucleotides when retrieving the gene sequence.

We note that GeneStitch does not explicitly consider the cycles that may be found in de Bruijn graphs, in order to use an efficient dynamic programming algorithm to solve the network matching problem: GeneStitch will traverse (randomly) through one of the cyclic paths (if present). In our tests, GeneStitch rarely encounters such cases, as gene sequences typically do not contain repeats.

### 2.2 Speeding the network matching process

The network matching algorithm described above aligns the reference sequence against the entire de Bruijn graph. The amount of time required for this process is linearly correlated to the number of nodes (representing contigs) in the graph and the lengths of the contigs. Accordingly, we implement two strategies to speed up the network matching procedure, given that a single gene will only span a small portion of the graph.

The first strategy is to use a similarity-based approach to constrain the search space in the de Bruijn graph for each reference gene sequence. First, we use BLAST to search all nodes (i.e. contigs) of the de Bruijn graph against the reference sequences with a relatively high *E*-value cutoff (currently set to 0.1). For each reference sequence, the node with the best alignment score will be used as the starting node to recruit more inbound and outbound nodes with BLAST hits. Considering that short contigs may be missed by the similarity search process (Wommack *et al.*, 2008), we allow the recruiting process to extend an additional *N* layers of inbound and outbound nodes without BLAST hits (*N* is set to 5). This process is repeated until no more nodes can be recruited. The included nodes (and the edges that connect them)—instead of the whole graph—then serve as the input graph for the network matching process.

The second strategy is to exclude intact genes found in the input contigs. We use FragGeneScan (Rho *et al.*, 2010) to predict fragmented genes as well as intact genes in all contigs, and then remove intact genes (defined as the predicted gene fragments that do not include the first or the last nucleotide of any contig) from the contigs prior to the network matching process, retaining only fragmented genes and intergenic regions adjacent to them. This preprocessing step greatly speeds the network-matching process.

### 2.3 Construction of gene graphs

Gene paths—each representing a (fragmented) gene—inferred from a de Brujin graph using homologous reference genes by the network matching algorithm described above may overlap with each other. These paths can be merged into a gene graph that represents a collection of homologous genes in a compact fashion.

To make sure that we generate gene graphs that consist of only homologous genes, three empirical criteria are applied when finding gene paths in the de Bruijn graph: (i) the optimal score of the alignment between the gene path and the reference gene is ≥50 (score threshold), (ii) the identity of the alignment is ≥60% (identify threshold) and (iii) the alignment covers at least 40% of the length of the reference sequence (gene coverage threshold). The identity threshold is set to 60%, since genes may not be very similar at the nucleotide level, especially if the reference genes are obtained from not-so-closely related species. Two gene paths sharing at least one contig are merged into a gene graph if the reference sequences used to infer the gene paths are highly similar (i.e. with identity ≥70%). We will further compare the merged gene graphs with other gene paths or gene graphs and merge them if they contain genes inferred from very similar reference genes. This merging process is performed between any two gene graphs until all pairs of graphs have been checked. Once the merging is completed, we will select—for each gene graph—its composite gene path with the highest network-matching alignment score as its representative sequence.

### 2.4 Extension of gene graphs

The network matching algorithm and the subsequent merging steps may leave out gene segments from the constructed gene graphs that are not sufficiently similar to the reference sequences. To make gene graphs complete, we will extend each gene graph by recruiting the inbound and outbound nodes of its contigs if they share similarities with the contigs already included in the graph. This process is repeated until no more nodes can be added. The algorithm is given as follows.


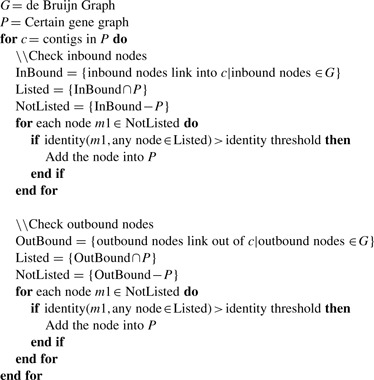


Currently, we set the identity threshold to 70% so that only very similar inbound and outbound contigs will be recruited into the gene graph.

### 2.5 Datasets and tools used

We implemented our algorithm in C++ and tested our program (named GeneStitch) on simulated datasets for a single genome and a dataset for an artificial microbial community.

We produced three test datasets of sequencing depths 6×, 13×, and 20× from the *Escherichia coli* str. K-12 substr. MG1655 genome (NC_000913) using Metasim software (Richter *et al.*, 2008). We used the 80 bp error model downloaded from the Metasim website to simulate Illumina reads of 80 bp with a 1% error rate. Genes from the *E. coli* HS (NC_009800), *Escherichia fergusonni* (NC_011740), and *Salmonella enterica* (NC_003198) were used as the references for GeneStitch.

The community dataset comprises sequencing reads obtained from an artificial microbial community with 10 mixed lab-cultured species (Morgan *et al.*, 2010). The main reason we chose this dataset (of 454 sequencing reads) as our test case is that we can directly evaluate the quality of the assembled genes because the genes and genomes of the species in the community are already known. Among the 10 species, 9 are either bacterial or archaeal, and 1 is eukaryotic (*Saccharomyces cerevisiae* S288C). We use genes from nine species as the reference gene sets, which are different at the species level (or higher level if species level is not available) compared with the bacteria or archaea species in the mock dataset. Table 1 lists the species we chose. We do not test on the eukaryotic genome because eukaryotic genes contain an intron–exon structure that our method is not currently designed for. To check for misassembly, we map assembled genes against the source genomes, using bwasw, provided by the BWA package (Li and Durbin, 2010). A gene is considered to be misassembled if it cannot be mapped, or maps to two or more locations in the genomes.

**Table 1.** The list of species contained in the mock dataset and corresponding species used as references in GeneStitch

Species in mock dataset	Reference species^a^
NC_002662 *Lactococcus lactis* subsp. *lactis* Il1403	NC_012984 *Lactobacillus plantarum* JDM1 (genus)
NC_008527 *L. lactis* subsp. *cremoris* SK11	NC_014724 *Lactobacillus amylovorus* GRL 1112 (order)
NC_008525 *Pediococcus pentosaceus* ATCC 25745	NC_008529 *Lactobacillus delbrueckii* subsp. *bulgaricus* ATCC BAA-365 (family)
NC_010999 *Lactobacillus casei* BL23	NC_014106 *Lactobacillus crispatus* ST1 (genus)
NC_008497 *Lactobacillus brevis* ATCC 367	NC_009513 *Lactobacillus reuteri* DSM 20016 (genus)
NC_008700 *Shewanella amazonensis* SB2B	NC_014012 *Shewanella violacea* DSS12 (genus)
NC_008095 *Myxococcus xanthus* DK 1622	NC_011891 *Anaeromyxobacter dehalogenans* 2CP-1 (family)
NC_008578 *Acidothermus cellulolyticus* 11B	NC_014666 *Frankia* sp. EuI1c (order)
NC_002607 *Halobacterium* sp. NRC-1	NC_013967 *Haloferax volcanii* DS2 (family)

^a^The taxonomic ranks in the parentheses indicate the lowest common taxonomy level shared between the reference species and the species in the mock dataset.

The SOAPdenovo assembler (Li *et al.*, 2010) is used to assemble both the simulated datasets and the artificial community dataset (we use k=31). The de Bruijn graph outputs from SOAPdenovo are used as inputs to GeneStitch. We use Δmatch = 1, Δmismatch = −2, Δ*g*_open = −3 and Δ*g*_ext = −1 in all our tests. Note that GraphStitch can work with any assembler that utilizes de Bruijn graphs.

## 3 RESULTS

### 3.1 GeneStitch improves gene assembly

We first test our algorithm on datasets simulated from only one genome (*E. coli* K-12) to show that reference genes from closely related (*E. coli* HS and *E. fergusonni*) or more distantly related species (*S. enterica*) can be used to improve gene assembly. We evaluate the performance of GeneStitch by both ‘gene coverage’, and the number of ‘complete genes’assembled. The gene coverage is defined as the average percentage of the annotated genes (in length) that are covered by the assemblies (e.g. a gene coverage of 100% means that full-length genes are assembled). An assembled gene is considered complete if it covers at least 90% of the actual gene, sharing at least 98% sequence identity.

The results are summarized in Table 2. Since GeneStitch is designed for assembling fragmented genes, we isolate the fragmented genes from the contigs either from the initial assembly or after various GeneStitch treatments and calculate the gene coverage for them (the statistics of all genes are also given). For all datasets, GeneStitch significantly improves the completeness of assembled genes as compared with initial assembly's genes (with higher gene coverage), and the number of complete genes, especially for the datasets with lower sequencing depths (6× or 13×). For example, for the dataset with 13× sequencing depth, SOAPdenovo alone assembled 2320 complete genes, and GeneStitch assembled 1070 more (i.e. a 46% improvement). Improvement is also observed, although less significant, for the dataset with 20× sequencing depth (which can already be assembled fairly well by SOAPdenovo with a gene coverage—for all genes in contigs—of 81%). These results demonstrate the ability of GeneStitch to link fragmented genes together and form longer genes.

**Table 2.** A summary of the GeneStitch results for *E. coli* K-12 at 6×, 13× and 20× sequencing depths

Sequencing depth	Reference	Genes/fragments*^a^*	Gene coverage*^b^*	Complete genes*^c^*	Complete gene ratio*^d^*	Misassembly rate
6×	—*^e^*	13 947 (14 149)*^f^*	26% (28%)*^f^*	572	14%	—
	*E. coli* HS	5365	62%	+318	21%*^g^*	0.3%
	*E. fergusonni*	4489	62%	+269	20%	0.5%
	*S. enterica*	3916	62%	+227	19%	0.2%
13×	—*^e^*	6642 (9158)*^f^*	33% (50%)*^f^*	2320	56%	—
	*E. coli* HS	4375	75%	+1070	82%	0.2%
	*E. fergusonni*	3664	75%	+932	78%	0.3%
	*S. enterica*	3212	75%	+824	76%	0.2%
20×	—*^e^*	1904 (3491)*^f^*	45% (81%)*^f^*	3264	79%	—
	*E. coli* HS	1960	75%	+461	90%	0.6%
	*E. fergusonni*	1484	76%	+418	89%	0.2%
	*S. enterica*	1261	75%	+349	87%	0.2%

*^a^*This column specifies the number of gene fragments in assembled contigs (the first row for each section) or the number of genes assembled by GeneStitch.

*^b^*Gene coverage reflects the completeness of assembled genes; a small value indicates that assembled genes are highly fragmented.

*^c^*This column lists the assembled genes or genes in contigs (the first row for each section) that are complete or almost complete (at least 90% of the entire length) as compared with the real genes. Additional complete gene numbers assembled by GeneStitch are highlighted by a ‘+’ sign.

*^d^* This column lists the ratio of completely assembled genes versus all annotated genes in the *E. coli* K-12 genome.

*^e^*This row lists the assembly results before applying GeneStitch.

*^f^* The two numbers indicate the statistics of fragmented genes and all genes (within parentheses) in contigs. See text for details.

*^g^*The ratio is calculated over all complete genes, including the ones assembled by SOAPdenovo and GeneStitch.

Another observation is that the improvement introduced by GeneStitch decreases with the taxonomic distances of the reference species, which is not surprising. Our tests, however, show that even when using distantly related species (e.g. *S. enterica*) as references, GeneStitch improved the quality of gene assembly. Overall these results demonstrate the power of GeneStitch, in which fragmented genes split into different contigs are assembled into longer gene fragments even if we use reference species of different genera (e.g. target species *E. coli* K-12 vs reference *S. enterica*).

We also examined the potential for misassembly in the assembled gene sequences by mapping the assembled genes against the *E. coli* K-12 genome. The proportions of misassembled sequences are very low for all three test datasets, indicating that GeneStitch introduces few misassemblies into single genome assemblies.

### 3.2 GeneStitch successfully identifies genes in a metagenomic dataset

**Fig. 2.**
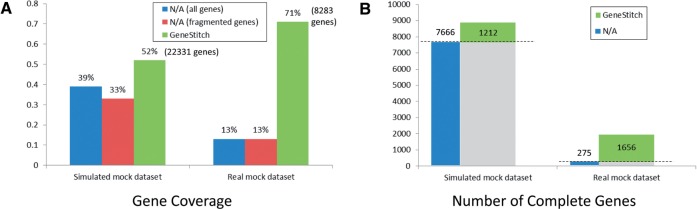
Improvement of gene assembly by GeneStitch for the simulated and real community datasets, as evaluated by gene coverage (A) and the number of complete genes (B)

We next tested GeneStitch with the artificial community dataset. Since the sequencing depth of the 454 dataset is not very high (2.86×) and contains a eukaryote organism, we also simulated a dataset with higher depth (9×) that included only the prokaryotic species from the dataset. The results are shown in Figure 2. Similar to the single genome cases, the gene coverage ratio for both the simulated and real metagenomic datasets increases (shown in Figure 2A), suggesting that GeneStitch is capable of assembling longer genes from the metagenomes. An intriguing observation is that even though there are fewer genes assembled from the real sequence dataset (8283 genes) as compared with the simulated dataset (22 331 genes), the gene coverage ratio of the assembled genes in the real dataset is actually higher after treatment with GeneStitch (71 versus 52%) .

The number of complete genes, as demonstrated in Figure 2B, also suggests that GeneStitch has the ability to produce complete genes from metagenomes. Besides the already complete genes in the contigs, GeneStitch is able to build 1212 and 1656 more complete genes from gene fragments. From the real dataset, GeneStitch assembled more than five times more complete genes than those in contigs. The reason that the number of complete genes assembled for the simulated dataset is less than that for the real dataset is that many complete genes are already well assembled for the simulated data due to its higher sequencing depth. On the other hand, the genes in the real dataset are mostly fragmented and are then recovered using GeneStitch. Nevertheless, the number of assembled genes for the simulated dataset (22 331 genes) is still higher than the real dataset (8283), suggesting that higher sequencing depth is still needed for ideal gene assemblies.

The misassembly rates for the genes assembled from the metagenomes are higher than those for single genomes. In total, 1109 genes (4.97%) and 165 genes (1.99%) are probably misassembled for the simulated and real dataset, respectively. Further analysis reveals that the majority of these genes (832 out of 1109 genes for simulated dataset and 37 of 165 genes for real dataset) can be mapped to exactly two homologous genes in the community: for example an assembled gene may consist of segments from two homologous genes and produce a chimeric sequence. Considering that these cases are sometimes unavoidable for metagenome assembly (and we call them ‘minor’ misassembles), especially when very similar genes from different species exist in the sample (there are two strains of *Lactococcus lactis*, namely *L. lactis* cremoris IL1403 and *L. lactis* cremoris SK11, in the mock dataset), the ‘severely’ misassembly rate is only 1.24 and 1.55% for the simulated and real datasets.

Below, we present two cases from the real community dataset, to demonstrate how we find the gene graph from the assembled de Bruijn graph.

### 3.3 Example gene graph No. 1

**Fig. 3.**
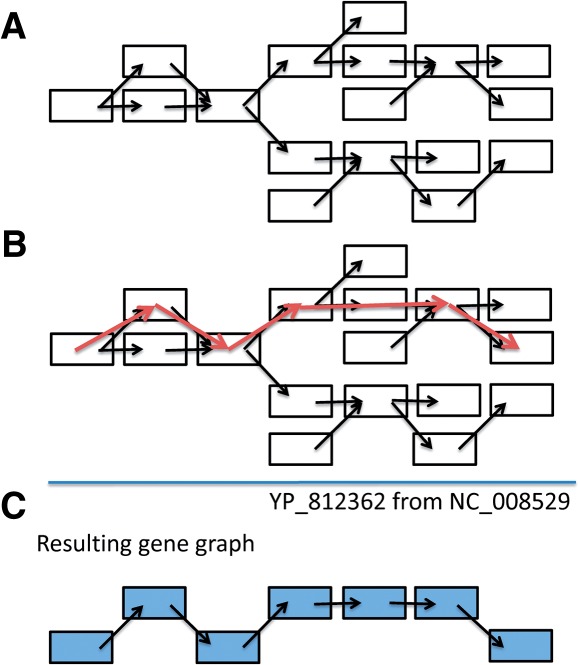
An example demonstrating the inference of a gene path from a connected component in the de Bruijn graph. The reference gene recruited by BLAST in this example is YP_812362. (A) In total, 17 nodes are present in this connected component. (B) The path found by GeneStitch using the reference gene. (C) The gene path

The first example demonstrates how a gene path can be inferred from a connected component in the de Bruijn graph with 17 nodes. Only one gene annotated as beta glucosidase, YP_812362 from the species *Lactobacillus delbrueckii* subsp. *bulgaricus* ATCC BAA-365, passes the threshold values and is detected in this example. The result is shown in Figure 3: the path with similarity to the reference gene contains seven nodes; no nodes can be further recruited into this connected graph, thus only seven nodes (contigs) covered by the path represent the gene graph, and the sequences in this path constitute the representative gene for this gene graph.

### 3.4 Example gene graph No. 2

**Fig. 4.**
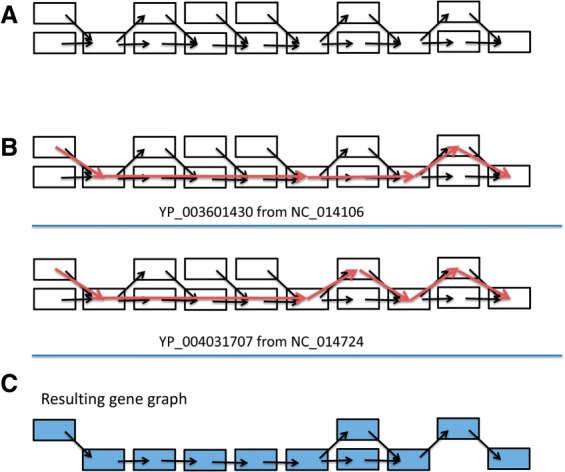
An example demonstrating the construction of a gene graph by merging gene paths. (A) only 19 nodes are shown in this figure for clarity (the actual component is larger). (B) Two paths are found by GeneStitch, using YP_003601430 and YP_004031707 as the reference genes. (C) The two paths are merged into a gene graph

This example demonstrates how we infer gene graphs by merging paths (or gene graphs). Figure 4A shows a connected component of the de Bruijn graph. Two reference genes, YP_003601430 from *Lactobacillus amylovorus* GRL 1112 and YP_004031707 from *Lactobacillus crispatus* ST1, can be recruited as reference genes to this graph. The identity between these two genes is 76%. From Figure 4B one can observe that the paths are very similar—only one branching node is different. Since the identity of the two reference genes is higher than the threshold (default 70%; see Section 2.3) and the two graphs are overlapping, these two graphs are merged into one gene graph, as shown in Figure 4C. The first assembled sequence, which has a higher score value (as well as a higher identity), is selected as the representative gene for this gene graph.

## 4 DISCUSSION

We present GeneStitch, which is based on a network matching algorithm, for inferring gene paths and gene graphs from the tangled de Bruijn graphs that result from assembly of metagenomic sequences. If we have prior knowledge of the taxonomic composition of a metagenomic dataset (e.g. through 16S rRNA gene profiling (Hamady *et al.*, 2008), or taxonomic analysis using shotgun sequences (Gerlach and Stoye, 2011)), we can use genes from the most closely related species available as references for GeneStitch, considering that GeneStitch benefits more by using the most similar gene sequences as the reference. However, in principle, we can use a general dataset of genes (e.g. microbial genes in the NCBI nr dataset) as reference genes in GeneStitch, if we have no prior knowledge of the taxonomic composition of a metagenomic sample.

For all tests that we performed, the application of GeneStitch greatly improves the assembly of genes, resulting in complete or nearly complete genes. The assembly of complete gene sequences is important because traditional metagenome sequencing projects are largely limited by the length of contigs and scaffolds, and small contigs are often difficult (if not possible) to use for subsequent functional analysis. We believe that our approach will increase the amount of information that can be gleaned from past and future genome and metagenome projects, by providing longer genes for analysis. We note that GeneStitch is able to improve the gene assembly even when only distantly related species are available as references, and when sequence depth is modest. This capability is especially important because sequenced bacterial or archaeal genomes are still limited and very closely related species (such as different strain of the same species) are not always available. GeneStitch greatly broadens the choice of reference species for gene annotation in metagenomic assemblies.

Our approach can be conceived as a gene predictor that works with de Bruijn graphs for assembly, instead of linear sequences. In this sense, GeneStitch is fundamentally different from current gene predictors including FragGeneScan (Rho *et al.*, 2010) and GLIMMER (Delcher *et al.*, 1999). Note that gene paths are fundamentally different from the directed acyclic graphs used to represent exons (as nodes) and their connectivity (the edges) in predictors for eukaryotic genes (Mathe *et al.*, 2002). We have also proposed a novel concept, the gene graph, to represent a collection of homologous genes in a metagenomic dataset. A gene graph may not include all similar (or homologous) genes in a metagenomic dataset, because we set the identity threshold to a relatively high value (e.g. 70%) in the process of constructing gene graphs. But it is not our goal to build comprehensive gene graphs; instead, we want to assemble metagenomic sequences into separate genes as long as we have strong evidence the assembled genes contain no misassemblies. We note that GeneStitch cannot help with the assembly of novel genes that lack similarity with known genes.

Although the gene graph is used to represent the cases where gene paths overlap with each other—a non-conventional way of representing genes—we argue that gene graphs can be considered as single units for downstream functional analysis of metagenomes. For example, we can attempt to get all real genes from the gene graphs by walking all potential paths in the gene graphs and select those supported by reads. This approach is used by the Trinity assembler to find all spliced isoforms and transcripts of recently duplicated genes from transcriptomes (Grabherr *et al.*, 2011). Another application would be functional prediction, we can search an unknown gene against all gene graphs and determine which gene graph is most similar to this gene, in order to determine its function.

Notably, other strategies have been used to improve metagenome assembly, for examples by merging assemblies from different assemblers or using the same assembler but with various parameter settings (Zimin *et al.*, 2008); by recruiting reads to fill in gaps between contigs using tblastn searches against reference genes as in the gene-boosted assembly approach (Salzberg *et al.*, 2008) and by assembling potential protein-coding reads at the peptide level as in the ORFome assembly approach (Ye and Tang, 2009). GeneStitch uses similarity between the genes included in a metagenomic dataset and reference genes available in a novel way and uses the matches between the de Bruijn graph assembly and the reference genes to improve the gene assembly. In principle, GeneStitch and other strategies to improve assembly can be combined to further improve the assembly of metagenomes.

## 5 CONCLUSION

We designed an approach to infer gene paths and gene graphs from a *de novo* assembled metagenomic dataset that each represents a gene or a single family of homologous genes. Each gene graph also generates a representative sequence that best represents the graph. We tested this approach on simulated datasets consisting of reads from one genome and the results are promising—longer genes are assembled and more intact genes are retrieved, and there are almost no misassembled genes. We also tested it with a dataset from an artificial microbial community and found that we again assembled more complete genes. We expect that gene graphs can be used to improve metagenome assemblies and that gene graphs will be a useful resource for the functional annotation of metagenomic samples.

## Footnotes

^1^Throughout this article, we consider the de Bruijn graph in which each simple path (a maximal directed path in the graph, in that all internal vertices have one incoming and one outgoing edge) is collapsed into a single node.
